# Pragmatic Management of EGFR-Mutant NSCLC After Progression on Osimertinib: Canadian Expert Perspectives

**DOI:** 10.3390/curroncol33080465

**Published:** 2026-08-05

**Authors:** Nathalie Daaboul, Jason S. Agulnik, Houda Bahig, Normand Blais, Marie-Ève Boucher, Nicole Bouchard, Marie-Hélène Denault, Patrice Desmeules, Pierre Olivier Fiset, Marie Florescu, Kevin Jao, Catherine Labbé, Magali Lecavalier-Barsoum, Carmela Pepe, Benjamin Shieh, Sophie Stock-Martineau, Nicolas Marcoux

**Affiliations:** 1 Centre Intégré de Cancérologie de la Montérégie, Hôpital Charles-Le Moyne, Greenfield Park, QC J4V 2H1, Canada; 2 Jewish General Hospital, McGill University, Montréal, QC H3A 0G4, Canadacarmela.pepe@mcgill.ca (C.P.); 3 Centre Hospitalier de l’Université de Montréal (CHUM), Université de Montréal, Montréal, QC H3T 1J4, Canada; 4 Hôtel-Dieu de Lévis, Lévis, QC G6V 3Z1, Canada; 5 Centre Hospitalier Universitaire de Sherbrooke, Université de Sherbrooke, Sherbrooke, QC J1N 3C6, Canada; 6 Institut Universitaire de Cardiologie et de Pneumologie de Québec–Université Laval (IUCPQ-ULaval), Québec City, QC G1V 4G5, Canadacatherine.labbe@criucpq.ulaval.ca (C.L.); 7 McGill University Health Centre, McGill University, Montréal, QC H3A 0G4, Canada; pierre.o.fiset@mcgill.ca (P.O.F.);; 8 Division of Medical Oncology and Hematology, Hôpital du Sacré-Coeur de Montréal, Montreal, QC H4J 1C5, Canada; 9 Hôpital Maisonneuve-Rosemont, Université de Montréal, Montréal, QC H3T 1J4, Canada; sophie.stock-martineau.med@ssss.gouv.qc.ca; 10 CHU de Québec–Université Laval, Université Laval, Québec City, QC G1V 0A6, Canada

**Keywords:** EGFR-mutated NSCLC, osimertinib, rebiopsy, MET amplification, oligoprogression, histologic transformation, Canada

## Abstract

The management of epidermal growth factor receptor-mutated (EGFRm) non-small cell lung cancer (NSCLC) after progression on osimertinib is becoming increasingly complex, particularly in Canada, where access to diagnostic testing and newer therapies remains uneven. Although treatment is evolving with regimens such as amivantamab−lazertinib (MARIPOSA/MARIPOSA-2) and osimertinib plus chemotherapy (FLAURA2), access to such treatments in first and second lines varies across provinces. This article provides a pragmatic Canadian perspective on post-osimertinib management informed by expert roundtable discussions, a focused clinician survey, and the contemporary literature. Key challenges identified include delays and barriers related to tissue biopsy, next-generation sequencing, timely immunohistochemistry in time to influence treatment decisions and access to novel therapies. These gaps reduce the ability to individualize care and often force clinicians to rely on platinum-pemetrexed therapy as the default systemic backbone, even when biologically relevant resistance mechanisms exist, highlighting that post-osimertinib care in Canada remains shaped as much by access and system constraints as by emerging evidence.

## Simple Summary

Osimertinib is a standard first treatment for a common type of lung cancer driven by mutations in the EGFR gene. When it stops working, deciding what to do next can be challenging in universal healthcare systems, where reimbursement of newer drugs and tumour testing might be inconsistent. This article brings together Canadian lung cancer specialists to share practical guidance on managing patients after osimertinib fails. Key issues include limited funding for newer combination therapies, delays in tumour biopsy and genetic testing, and the frequent need to rely on chemotherapy when better-targeted options are unavailable or inaccessible.

## 1. Introduction

Lung cancer is biologically and clinically heterogeneous, and non-small cell lung cancer (NSCLC) with mutations in the epidermal growth factor receptor (EGFR) gene represents a distinct molecular subtype whose prevalence varies across regions and populations. The proportion of patients with EGFR-mutated (EGFRm) disease can exceed 40–50% in some Asian cohorts, while in Western populations it is usually in the 10–20% range, with important differences according to sex, smoking status, and ethnicity [1,2]. In Canada, the prevalence of EGFRm disease is approximately 20%, varying among populations [3,4,5]. This reality underscores the importance of pragmatic, locally adapted guidance that reflects both the biology of EGFRm disease and the organizational and reimbursement environment in which care is delivered.

Following its approval, osimertinib, a third-generation EGFR tyrosine kinase inhibitor (TKI), rapidly became the standard first-line treatment for patients with advanced NSCLC with classic sensitizing mutations in EGFR. Compared with earlier-generation EGFR TKIs, osimertinib provides superior progression-free survival (PFS), improved central nervous system (CNS) control, and a generally favourable tolerability profile, leading to its broad adoption in routine practice as first-line therapy in EGFRm NSCLC [6,7,8]. However, as with other targeted therapies, resistance to osimertinib occurs, and disease progression is highly heterogeneous with respect to timing, pattern of relapse, and underlying resistance mechanisms [9,10].

Several international expert groups, including the European Society of Medical Oncology (ESMO) consensus panel, have proposed algorithms for managing resistance to EGFR TKIs, including osimertinib, and emphasize the importance of repeat biopsy, molecular profiling, and multidisciplinary evaluation [11,12]. Nonetheless, these guidelines cannot fully account for regional differences in diagnostic pathways, drug access, and healthcare organization. Across Canada, variability in availability of comprehensive next-generation sequencing (NGS), access to MET-directed agents and antibody–drug conjugates (ADCs), and constraints related to publicly funded care all influence what can realistically be offered to patients [4,13]. Despite recent advances, there is still a need for detailed, Canada-specific perspectives to inform the management of patients with advanced EGFRm NSCLC progressing on osimertinib.

## 2. Methods

The objective of this work is to provide a pragmatic Canadian perspective on the management of EGFRm NSCLC after progression on osimertinib. These perspectives and suggested approaches are informed by a focused literature review, expert roundtable discussions, and a survey designed to capture contemporary practice perspectives and implementation challenges [4,10,11]. This work is intended as a pragmatic position paper rather than a formal consensus statement or clinical practice guideline. Its purpose is to place published evidence alongside Canadian expert experience, highlight practical healthcare-system challenges, and inform—not prescribe—clinical decision-making.

To inform this expert perspective, two round-table discussions (attended by 15 and 17 experts) and four small group sessions (each attended by 4–7 experts) took place starting in 2022 as an ongoing process, involving oncologists, respirologists, radiation oncologists, and pathologists specialized in treating lung cancer. Experts were chosen to represent the multidisciplinary team and to include all academic centres and key opinion leaders from the province. The conversations aimed to initiate a broad discussion on the current challenges and advancements in the treatment and management of EGFRm NSCLC, progression after osimertinib, oligoprogression, MET amplification, and other mutations/transformations, including mixed progression. The group felt that the conversations during the roundtables were insightful and could help inform decision-making, so further steps were sought to share these insights with the broader oncology community. To validate and further refine the discussion points, a series of email exchanges were conducted. Specific questions were posed to the experts to clarify and validate the insights gathered during the roundtable and small group discussions.

Because the field continued to change after the initial discussions, the group felt a survey reflecting changes in treatment would help capture current practice perspectives and sharpen the manuscript’s practical relevance. The survey was developed from themes and unresolved practical questions identified during the roundtable process. Questions were designed to reflect key decision points along the patient journey. The survey included questions on first-line treatment expectations, rebiopsy and molecular testing at progression, management when no actionable resistance mechanism is identified, oligoprogression, MET-driven resistance, histologic transformation, emerging therapies and ongoing trials.

**Figure 1:**
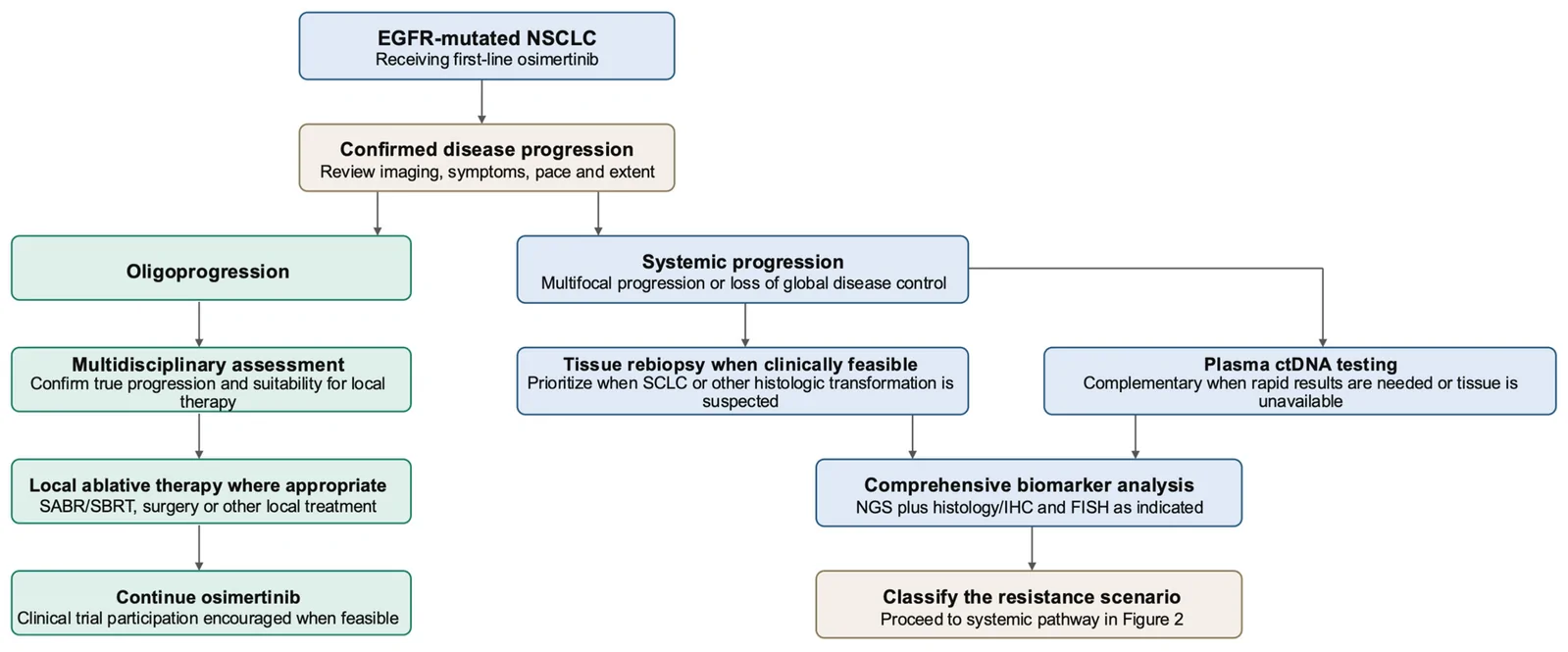
Reassessment and management pathways after progression on first-line osimertinib. Green boxes indicate evidence-supported local-treatment strategies; blue boxes indicate diagnostic assessment; beige boxes indicate classification or transition points. ctDNA, circulating tumour DNA; FISH, fluorescence in situ hybridization; IHC, immunohistochemistry; NGS, next-generation sequencing; SABR, stereotactic ablative radiotherapy; SBRT, stereotactic body radiotherapy; SCLC, small-cell lung cancer.

**Figure 2:**
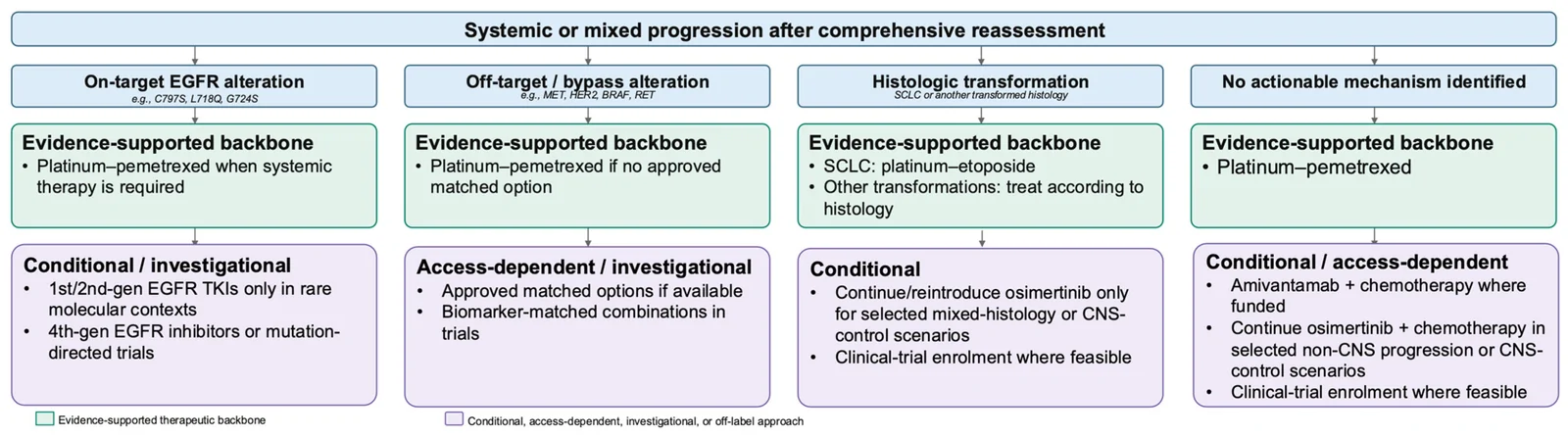
Systemic management pathways after comprehensive reassessment. Green boxes identify evidence-supported therapeutic backbones. Purple boxes identify investigational, off-label, or access-dependent approaches that may be considered in selected patients, ideally following expert multidisciplinary review or within a clinical trial. The pathways are intended as practical decision support rather than prescriptive treatment recommendations. ADC, antibody–drug conjugate; CNS, central nervous system; EGFR, epidermal growth factor receptor; HER2, human epidermal growth factor receptor 2; MET, mesenchymal–epithelial transition; RET, rearranged during transfection; SCLC, small-cell lung cancer; TROP2, trophoblast cell-surface antigen 2.

The survey (Supplementary Table S1) was written in French and distributed in February 2026 to 13 oncologists and respirologists who specialize in treating lung cancer and were involved in the initiative (and included as authors). Two radiation oncologists and two pathologists were also invited to inform questions specific to radiation and biomarker testing, respectively, and to provide expertise on this manuscript. This survey was intended to inform practical interpretation and not to serve as a formal Delphi or voting-based consensus process. Survey percentages are presented as descriptive findings reflecting the participating clinicians’ practice perspectives, not as consensus thresholds or evidence-graded recommendations. Published evidence and existing guidelines are cited separately and used to contextualize these perspectives. Figure 1 and Figure 2 further distinguish evidence-supported backbones from conditional, access-dependent, or investigational options.

A focused narrative literature review was conducted to contextualize the survey findings. Sources included PubMed-indexed publications and ClinicalTrials.gov records, updated through 31 May 2026, identified using combinations of terms related to EGFRm NSCLC, osimertinib resistance or progression, rebiopsy, tissue and liquid biopsy, MET amplification, histologic transformation, oligoprogression, and post-osimertinib therapies or clinical trials. Publications were selected for direct relevance to issues raised during the roundtables or survey and to contemporary Canadian practice. This was a focused narrative review rather than a systematic review; no formal risk-of-bias assessment or quantitative evidence synthesis was performed, and the review was not intended to be exhaustive [14,15,16].

## 3. Evolving First-Line Treatment

The first-line treatment landscape for EGFRm NSCLC is evolving rapidly following the results of FLAURA2 and MARIPOSA. Single-agent osimertinib remains a standard first-line treatment for many patients in Canada, but two newer combination strategies have demonstrated improved outcomes. In FLAURA2, the addition of platinum-pemetrexed chemotherapy to osimertinib improved PFS, enhanced CNS control, and prolonged overall survival (OS) by 10 months compared with osimertinib alone [8,14]. Similarly, in MARIPOSA, a combination of amivantamab, an anti-EGFR and anti-MET bispecific monoclonal antibody, and lazertinib, a third-generation TKI, was studied in first-line therapy. Amivantamab plus lazertinib significantly improved both PFS and OS compared with osimertinib monotherapy [15,16]. Combination therapy may be particularly relevant for patients with poor prognostic features, for whom osimertinib monotherapy may be less likely to provide durable systemic control [8,17,18,19]. These include patients with CNS metastases, high tumour burden or extensive metastatic disease, detectable baseline circulating tumour DNA (ctDNA), and co-occurring alterations such as TP53 mutations. In such patients, first-line intensification with osimertinib plus platinum-pemetrexed or amivantamab–lazertinib may improve depth and durability of response compared with osimertinib alone, although treatment selection must be balanced against toxicity, patient fitness, route of administration, quality-of-life considerations, and access [8,14,15,16].

In current practice, osimertinib monotherapy remains the foundational first-line option for EGFRm NSCLC, although treatment selection is increasingly individualized. When asked what first-line therapy they expect their patients to receive, respondents anticipated broader use of osimertinib with or without chemotherapy (FLAURA2), while most respondents expected that fewer patients would receive amivantamab−lazertinib (MARIPOSA). This pattern is consistent with osimertinib as the established benchmark in the first-line setting, as well as the current state of public reimbursement. The FLAURA2 and MARIPOSA regimens are approved in Canada, although funding for amivantamab-based regimens varies across provinces [17,18,19]. However, not all treatment decisions are access-based but depend on other factors as well, such as toxicity and patient preference. For example, in FLAURA2, rates of grade 3 adverse events (AEs) as well as AEs leading to discontinuation were approximately doubled with chemotherapy plus osimertinib compared to osimertinib alone [8,14]. However, as platinum-based chemotherapy is an established backbone of NSCLC treatment, clinicians are familiar with management of FLAURA2-based AEs. With MARIPOSA, the rate of grade ≥3 AEs was higher with amivantamab−lazertinib (80% of patients) versus osimertinib (53%); a higher rate of AEs may deter some patients from such regimens. Furthermore, as a novel combination, clinicians may take more time to become familiar with its management and use it more broadly [15]. Survey responses indicated that first-line osimertinib monotherapy would be preferred based on patient preference for easier administration and toxicity management, as well as for quality-of-life considerations and low tumour burden. This indicates a pragmatic approach in which first-line intensification is considered for fit patients with more aggressive disease features, whereas osimertinib monotherapy remains a reasonable option for patients for whom a simpler, better-tolerated oral regimen is preferred.

## 4. Second-Line Management

### 4.1. Treatment Selection and Sequencing

Along with evolving treatment selection in first-line treatment, second-line treatment is also changing. In MARIPOSA-2, amivantamab plus chemotherapy or amivantamab−lazertinib plus chemotherapy demonstrated significantly improved PFS versus chemotherapy alone in patients who progressed on osimertinib; second interim OS data showed a favourable OS trend versus chemotherapy alone [16,20]; however, final OS data are pending. Accordingly, when asked what regimen they would favour after progression on FLAURA2, assuming no access restrictions, the more frequent responses were MARIPOSA-2 (54%), followed by clinical trial participation (38%), with a small minority favouring platinum-doublet rechallenge with continued osimertinib. The Canadian Drug Agency (CDA) has recommended reimbursement of amivantamab-based regimens (MARIPOSA-2), allowing possible use in second-line treatment. However, reimbursement may not be available in all provinces, suggesting that first-line choice could be made with later treatment options in mind. At present, however, it is important to recognize that post-FLAURA2 or post-MARIPOSA sequencing is not yet well defined. Nevertheless, the availability of amivantamab-based regimens in first- but not second-line treatment is an important consideration when choosing treatment regimens.

### 4.2. Reassessment at Progression: Rebiopsy and Testing

Acquired resistance to osimertinib is biologically heterogeneous; thus, identifying the mechanism through biopsy directly informs subsequent management, including eligibility for targeted combinations, clinical trials, or histology-specific therapy [11,21]. Testing is conducted using either tissue biopsy, plasma circulating tumour DNA (ctDNA; liquid biopsy), or both [11]. Tissue biopsy allows histopathological diagnosis to identify small-cell lung cancer (SCLC) transformation or another histologic transformation and provides material for molecular profiling. Tissue-based assays such as immunohistochemistry and FISH can be used in addition to NGS to identify amplifications and protein overexpression at the sampled tumour site. Liquid biopsy has the advantage of capturing tumour heterogeneity but has limited sensitivity compared with tissue testing [22]. Broadly, resistance mechanisms after osimertinib fall into three groups: on-target EGFR alterations; off-target or bypass pathway alterations; and histologic transformations [11,21,23]. A substantial proportion of patients have no clearly identifiable actionable mechanism even after testing, which further supports using the broadest feasible molecular work-up whenever progression occurs [11,21,23].

Tissue biopsy remains the gold standard, particularly when SCLC transformation or another histologic transformation is suspected, because it provides both morphologic confirmation and material for molecular testing. Histologic transformation cannot be diagnosed by plasma ctDNA alone. However, tissue biopsy is invasive, may not always be feasible, and can delay treatment decisions; adequate material might not always be obtained [24]. Liquid biopsy offers a less invasive alternative that can be repeated more easily and may better capture tumour heterogeneity, but it has lower sensitivity for certain resistance mechanisms, particularly amplifications and fusions, and cannot establish transformation [25,26,27]. Tissue and plasma ctDNA testing are therefore complementary, with tissue prioritized when clinically feasible—especially when the clinical pattern raises concern for SCLC transformation—and liquid biopsy is used when rapid reassessment is needed, or tissue sampling is not feasible or has failed [25,26,27,28].

Our survey indicated that rebiopsy is strongly favoured after progression on osimertinib. Most respondents either favoured combined tissue and liquid biopsy (38%) or only tissue biopsy when possible (38%). Another 23% would biopsy only in selected cases. Only 15% routinely requested specific testing for mesenchymal–epithelial transition (MET) amplification alongside NGS, while 85% did not. Even with the availability of options such as amivantamab-based regimens as evaluated in MARIPOSA-2—which did not require fresh tumour biopsy—most respondents (62%) would still request rebiopsy at progression, whereas 38% felt that increased availability of MARIPOSA-2 might reduce the need for biopsy. This ongoing need to distinguish between histologic transformation, MET-driven resistance, and other potentially actionable pathways is consistent with expert recommendations, which also support repeat molecular assessment at progression on EGFR TKI therapy whenever feasible [11,12,13,29,30].

A key barrier expressed by most clinicians was biopsy delays and turnaround times: 69% said that delays in obtaining the biopsy and its results can create a barrier to ordering a biopsy. Reported turnaround times for biomarker/NGS results varied: 38% reported 2 weeks or less, 23% reported 3 weeks, and 38% reported 4 weeks or more. Thus, from a practical perspective, the main challenge is not whether rebiopsy is valuable, but whether it can be performed quickly and broadly enough to influence care. Respondents also reported variable turnaround times and limited routine testing for MET amplification, highlighting the gap between the ideal diagnostic pathway and what is consistently achievable in practice. Across Canada, access to MET FISH is shaped by provincial testing and reimbursement pathways. Because implementation of molecular assays is often linked to recognized or reimbursed targeted therapy indications, MET FISH may not be routinely available when MET-directed therapy for acquired MET amplification after osimertinib is not funded, contributing to administrative delays and regional variability in resistance testing. Overall, the published evidence and survey findings position rebiopsy as a high-priority consideration at clinically meaningful progression, using tissue, plasma, or both depending on feasibility, suspected biology, and local access [11,31].

Although rebiopsy and molecular profiling may reveal newly acquired alterations associated with osimertinib resistance, many therapies directed against these mechanisms remain investigational; therefore, clinical trial participation should be considered whenever feasible after progression. Respondents (77%) were satisfied with the number and variety of EGFRm lung cancer clinical trials open at their respective centres, though 23% felt there should be more. This is particularly important as many patients might only have access to newer therapies through clinical trials. A summary of clinical trials recruiting in Canada for this population (as of May 2026) is shown in Table 1.

On-target EGFR resistance mutations such as C797S, L718Q, and G724S are clinically important but generally do not yet have approved targeted therapies in the post-osimertinib setting, and management remains largely investigational [32,33]. Histologic transformation is generally actionable, but mainly in a histology-directed rather than targeted manner; for example, small-cell transformation typically shifts treatment toward platinum−etoposide-based therapy, while other transformations are treated according to the transformed histology [32,33]. Among the major categories of acquired resistance after osimertinib, the most clearly actionable with approved therapies are off-target/bypass alterations; again, this is highly dependent on the specific mechanism. Recent reviews describe resistance mechanisms and emerging therapeutic approaches to such mechanisms in detail [21,32,33].

## 5. On-Target and Off-Target Mutations

### 5.1. On-Target EGFR Mutations

On-target EGFR resistance after osimertinib is driven by secondary alterations in the EGFR kinase domain that reduce drug binding, most commonly C797S, and less frequently L718Q, G724S, G796S, and related mutations near the ATP-binding pocket [16,21,22]. These alterations account for roughly 10–15% of acquired resistance events after first-line osimertinib and are clinically important because they confirm continued EGFR dependence. However, their therapeutic implications remain uncertain because treatment sensitivity may depend on the original activating EGFR mutation, T790M status, allelic configuration, and coexisting resistance mechanisms. No targeted therapies are currently approved specifically for post-osimertinib on-target EGFR resistance, and clinical evidence supporting currently available first- or second-generation EGFR TKIs is limited and highly dependent on the specific mutation and broader molecular context. Management therefore remains investigational, and enrolment in relevant clinical trials should be considered where available [21,32,33].

### 5.2. Off-Target

Off-target resistance to osimertinib comprises bypass pathway alterations that maintain tumour signalling despite continued EGFR blockade [21,32,33]. These include MET amplification, HER2 amplification or mutation, KRAS or BRAF mutations, PIK3CA alterations, and acquired ALK or RET fusions. Among post-osimertinib resistance mechanisms, these alterations are the most likely to be considered actionable; however, most targeted therapies approved in Canada were developed for NSCLC in which the alteration is the primary oncogenic driver, not specifically for acquired resistance after osimertinib.

The most recent development with respect to off-target resistance mechanisms is the results from HERTHENA trials examining anti-HER3 therapy in post-EGFR TKI EGFRm NSCLC. Patritumab deruxtecan showed clinically meaningful activity in phase II HERTHENA-Lung01, but the subsequent phase III HERTHENA-Lung02 study did not demonstrate meaningful benefit, with only a modest gain in PFS and no improvement in OS. Accordingly, HER3-directed therapy cannot currently be considered an established standard after osimertinib [34,35].

### 5.3. MET-Driven Resistance and Anti-MET Strategies

MET amplification remains one of the most important and best-studied off-target acquired resistance mechanisms in EGFRm NSCLC, reported in up to 24% of patients [32,33]. It is important to distinguish MET amplification from MET point mutations or MET exon 14 skipping, which represent different biological entities and have different therapeutic implications [32].

MET amplification usually emerges as a bypass resistance mechanism that allows tumour signalling to continue despite EGFR inhibition. Overall, the ideal testing approach for MET amplification involves initial screening with NGS, followed by FISH, which measures gene copy numbers, as confirmation [36]. However, both these methods are limited by variations in tumour content, which can affect detection accuracy. NGS may be suboptimal for detecting amplifications due to the requirement of large amounts of DNA and complex analyses [36]. On the other hand, FISH is limited by copy number thresholds, which can vary and require validation based on standardized thresholds. Liquid biopsy techniques are particularly useful for assessing tumour heterogeneity and monitoring disease progression over time, although further validation is needed for their use in detecting MET amplification [26].

From a therapeutic standpoint, dual EGFR/MET inhibition is particularly compelling in MET-amplified resistance because the original EGFR driver usually persists, while MET acts as a bypass pathway, allowing continued tumour signalling despite EGFR blockade. This provides a strong biologic rationale for maintaining EGFR inhibition while simultaneously targeting MET. Importantly, however, not all EGFR/MET-directed strategies were developed specifically for biomarker-selected patients with confirmed MET amplification. For example, amivantamab-based approaches such as MARIPOSA-2 were studied more broadly in patients with EGFR-mutant NSCLC progressing on osimertinib and were not restricted to those with documented MET-amplified resistance. In this sense, amivantamab-based therapy may be viewed as a more mechanism-agnostic EGFR/MET strategy, whereas other combinations have been developed more specifically for patients with confirmed MET-driven resistance.

Among these more biomarker-directed strategies, savolitinib plus osimertinib has shown particularly encouraging results. In the phase II SAVANNAH study, this combination demonstrated clinically meaningful and durable activity in patients with MET overexpression and/or amplification after progression on osimertinib [37]. This approach is now being tested in the phase III SAFFRON trial (not to be confused with the SAFFRON-301 trial investigating sitravatinib plus tislelizumab), which compares savolitinib plus osimertinib with platinum-based chemotherapy [38]. The findings from SAVANNAH are complemented by the randomized phase III SACHI trial, which reported improved PFS versus platinum-doublet chemotherapy in patients with EGFRm, MET-amplified disease after first-line EGFR TKI therapy [39]. Similarly, in INSIGHT-2, the combination of osimertinib plus tepotinib demonstrated promising activity and acceptable safety in patients with MET amplification after first-line osimertinib, supporting a chemotherapy-sparing oral strategy in this population [40]. These MET-directed combinations, including savolitinib plus osimertinib, have not been approved in Canada, underscoring the persistent gap between a strong biological and clinical rationale and routine access in practice.

Finally, telisotuzumab vedotin (Teliso-V) is an antibody−drug conjugate (ADC) that targets c-Met protein overexpression via telisotuzumab, which is conjugated to vedotin. Thus, Teliso-V seeks out tumour cells with high c-MET protein expression and delivers the cytotoxic payload to those cells [41]. In a phase Ib study of tuzumab vedotin plus osimertinib after progression on prior osimertinib, the combination achieved favourable response rates and PFS, with a manageable but notable toxicity profile that included peripheral neuropathy and edema [41]. This combination remains investigational.

Our survey results suggest that clinicians viewed MET-driven resistance as a setting in which practice is strongly shaped by both biological rationale and real-world access constraints. When asked which anti-MET strategy seemed most promising after osimertinib, respondents were divided between MARIPOSA-2 (54%) and savolitinib plus osimertinib (46%), while no respondents selected tepotinib plus osimertinib or telisotuzumab vedotin plus osimertinib as the most promising option. At the same time, respondents noted that access to anti-MET combinations in real-world practice was very limited: 85% reported that they had not been able to obtain anti-MET targeted therapy combined with osimertinib outside a research protocol, while 8% had obtained access through compassionate use programmes and 8% reported out-of-pocket payment. These responses are consistent with the broader Canadian reality, in which identifying MET amplification does not necessarily mean that a MET-directed combination is readily obtainable in routine care.

Overall, these data support MET amplification as one of the most actionable post-osimertinib resistance states biologically, even if Canadian access to anti-MET plus osimertinib combinations remains limited in practice [10,11].

### 5.4. No Actionable Resistance Mechanism Is Identified

Even after rebiopsy and sequencing, many patients have no identified actionable resistance mechanism after progression on osimertinib. In this scenario, respondents’ preferred option was MARIPOSA-2 (62%), followed by continuation of osimertinib with platinum-doublet chemotherapy (23%) and clinical trial enrollment (15%).

However, in routine practice, platinum-pemetrexed therapy remains the conventional systemic backbone after osimertinib when no targetable resistance mechanism is found, most likely due to restricted access to amivantamab in second-line treatment in some jurisdictions and to the lack of other available options in this setting. In randomized post-EGFR TKI trials, outcomes with chemotherapy alone have been modest, with median PFS generally around 4–5 months and median OS approximately 15–16 months, underscoring the limited durability of disease control in this population. The limitations of this approach are important: efficacy is constrained, treatment is fully systemic rather than biologically tailored, and conventional platinum-pemetrexed therapy has limited intrinsic CNS activity, which is clinically relevant in EGFRm NSCLC where brain metastases are common and ongoing intracranial control may be a priority [29,42].

Experience with the MARIPOSA-2 regimen was generally favourable: 77% of respondents reported having used the regimen and considered it useful and relevant, whereas 15% felt the benefit was more limited and 8% had not used it. This suggests that clinicians perceive meaningful real-world utility of this regimen despite access limitations, and would want to prescribe it, if possible.

Clinicians also strongly supported maintaining access to osimertinib after progression, with 62% responding yes and 38% responding maybe (for selected patients). The use of osimertinib for preservation of CNS control was noted as a priority. This is supported by the results of the COMPEL phase III trial [43], which demonstrated improved PFS and longer OS with osimertinib plus chemotherapy compared with placebo plus chemotherapy after non-CNS progression on osimertinib in first line.

Taken together, the evidence and survey responses suggest a practical sequence of considerations in patients without an actionable resistance mechanism: rebiopsy when feasible, platinum-pemetrexed as the current therapeutic backbone, selective continuation of osimertinib in carefully chosen patients, and consideration of amivantamab-based therapy where available and reimbursed.

## 6. Histologic Transformation to SCLC

Approximately 3–10% of acquired resistance to EGFR TKIs is associated with transformation to SCLC [22]. Management remains challenging and is supported mainly by retrospective data [44,45]. In our survey, respondents most often favoured platinum–etoposide alone (54%), while a substantial minority (46%) favoured platinum–etoposide plus osimertinib; platinum–etoposide plus immunotherapy was not used. These preferences are consistent with the available literature, in which platinum–etoposide remains the best-supported treatment backbone after transformation, whereas the benefit of continuing osimertinib remains uncertain and is generally considered on a case-by-case basis, particularly when there is concern for persistent EGFR-driven disease, mixed histology, or maintenance of CNS control [22,23,46]. By contrast, the role of checkpoint inhibitors remains unclear and has not shown convincing benefit in the limited retrospective series available [22,23]. Overall, these data support platinum–etoposide as the default approach, with selective continuation of osimertinib in carefully chosen patients.

## 7. Immunotherapy as Salvage

Although immunotherapy has demonstrated dramatic improvements in other tumour types, this therapeutic strategy has shown mostly limited benefits in EGFRm NSCLC. Results from the phase III KEYNOTE-789 trial (pembrolizumab plus pemetrexed-platinum chemotherapy) or phase III CheckMate 722 (nivolumab plus platinum-based chemotherapy) did not demonstrate significantly improved PFS or OS compared with chemotherapy alone in EGFRm metastatic NSCLC after progression on EGFR TKIs [47,48]. However, there is some evidence that the addition of vascular endothelial growth factor (VEGF) inhibitors to immunotherapy might be beneficial in the post-osimertinib population. For example, in an exploratory subgroup analysis of EGFRm patients in IMpower150, a signal of benefit was seen for atezolizumab + bevacizumab + carboplatin + paclitaxel compared with bevacizumab chemotherapy alone [49]. The phase III HARMONi-A trial provided promising results, showing significantly improved PFS with the VEGF x PD-1 bispecific antibody ivonescimab plus chemotherapy compared with chemotherapy alone [50]; however, ivonescimab is not yet approved in Canada. In our survey, most respondents (69%) felt ivonescimab was promising but wanted to wait for ongoing studies; a total of 23% did not think it was relevant because better options exist, and only 8% wanted access immediately.

Overall, survey responses aligned with current evidence in suggesting that immunotherapy, if used at all, should be reserved for selected salvage situations rather than standard practice: 38% of respondents indicated that they would offer immunotherapy regardless of PD-L1 expression, 23% would consider it mainly in patients with high PD-L1 expression (≥50%), and 38% stated that they do not use immunotherapy in EGFRm disease, even as a last resort. Immunotherapy is generally viewed as a late-line, highly selective option rather than a standard component of post-osimertinib management [13,29].

## 8. Management of Oligoprogression

Oligoprogression is a state where a limited number of metastatic sites show progression while other metastatic sites remain controlled under systemic therapy. This concept is pivotal in the era of targeted therapy and immunotherapy, where isolated metastatic sites may develop resistance and grow, while most other sites continue to respond to treatment. Most commonly, oligoprogression is defined by three to five progressing metastatic lesions [51]. However, the exact number can vary based on expert consensus and clinical context, including the tumour type, treatment goals, and available therapeutic options. The European Society for Radiotherapy and Oncology (ESTRO) and the American Society for Radiation Oncology (ASTRO) published a consensus document stating that oligoprogression generally involves one to five metastatic lesions [52].

Imaging plays a crucial role in the thorough evaluation of oligoprogression. PET scans are strongly recommended, although accessibility and interpretation may be limitations. Brain magnetic resonance imaging (MRI) is also recommended, and some experts recommend endobronchial ultrasound (EBUS) depending on imaging results. Before initiating local therapy, a thorough evaluation is essential to confirm the presence of oligoprogression. Lung function tests should be repeated, especially for smokers, when thoracic radiotherapy is considered. Moreover, several factors must be considered on a case-by-case basis. These should include anticipated local control at progressing sites, potential toxicities, performance status, symptomatology, significance of changes observed, and imaging delays impacting treatment start.

Management strategies for oligoprogression can differ depending on the location of the progressing lesions, such as thoracic, extra-thoracic, or CNS, and include the use of systemic therapy (including osimertinib and chemotherapy), local radiotherapy, and surgery. The addition of local radiotherapy to sites of oligoprogression has been shown to allow patients to remain on systemic therapy longer [53] and is recommended in guidelines [13]. This approach may include stereotactic ablative radiotherapy (SABR) or other dose-intensive accelerated/hypo-fractionated chemoradiotherapy regimens if SABR is not possible [13]. The ongoing SUPPRESS-NSCLC study aims to evaluate SABR in continuation of current systemic therapy versus standard of care in patients with oligoprogressive NSCLC [54].

For CNS oligoprogression, it is essential to assess whether the patient is exhibiting symptoms of brain metastases. Collaboration with a radiation oncologist is critical to determine which radiation modality is needed; radiosurgery is usually favoured over whole-brain radiotherapy when the number of metastases is limited. Another important consideration is whether to add chemotherapy for patients experiencing CNS progression while on osimertinib.

Available evidence suggests that selected patients may benefit from continuing osimertinib, while receiving local radiotherapy for oligoprogressive disease [53]. Patients with oligoprogression who remain on osimertinib showed longer PFS; however, it is important to note that benefit was not observed for patients with broader systemic progression [55]. Finally, a prospective phase II study evaluated osimertinib rechallenge after local ablative therapy to oligoprogressive sites. Although PFS benefit was not observed across the entire study population, meaningful improvements in PFS were observed for patients with ctDNA-negative minimal residual disease of the EGFRm clone prior to osimertinib rechallenge [56]. There is still no definitive study specifically demonstrating the superiority of continuing osimertinib over an immediate systemic treatment switch.

All respondents indicated that they would prefer to maintain osimertinib as long as possible and use radiotherapy as needed; no respondent indicated that they would be more inclined to change systemic treatment if they could obtain amivantamab or another systemic therapy. This aligns with currently available evidence as well as being cost-effective, an important consideration in a universal healthcare setting.

Overall, oligoprogression represents a distinct clinical scenario requiring tailored management strategies. Management may involve a combination of systemic and local therapies, individualized according to the patient’s overall condition, sites of progression, anticipated toxicity, and treatment goals. Multidisciplinary collaboration and clinical trials are essential to advancing the understanding and management of oligoprogression in lung cancer.

## 9. Emerging Therapies and Future Directions

The post-osimertinib treatment landscape is likely to continue expanding through antibody–drug conjugates, resistance-directed combinations, and newer EGFR inhibitors. These developments create opportunities but also reinforce the need for prospective sequencing evidence and implementation strategies that account for Canadian diagnostic and reimbursement pathways.

New anti-TROP2 ADCs were generally viewed positively. For datopotamab deruxtecan (Dato-DXd), 62% of respondents felt it looked promising after osimertinib plus chemotherapy and would like access now, while 38% preferred to wait for ongoing studies. Dato-DXd was evaluated in the phase III TROPION-Lung01 trial versus docetaxel in previously treated advanced/metastatic NSCLC; it improved PFS in the overall population, especially in nonsquamous histology, and showed a clinically meaningful but not statistically significant OS trend, with a more favourable grade ≥3 toxicity profile than docetaxel, although stomatitis and interstitial lung disease/pneumonitis remain important risks [57,58]. A pooled analysis of TROPION-Lung01 and TROPION-Lung05 also showed an encouraging signal in patients with EGFRm NSCLC who had progressed on EGFR TKIs [57]. In June 2025, the US Food and Drug Administration granted accelerated approval for Dato-DXd in adults with locally advanced or metastatic EGFRm NSCLC after prior EGFR-directed therapy and platinum-based chemotherapy [59]. In Canada, datopotamab deruxtecan remained under regulatory review as of June 2026 and was not yet an approved or reimbursed post-osimertinib option [60].

Conversely, for sacituzumab tirumotecan (sac-TMT; MK-2870/SKB264), 62% preferred to wait for more data, while 38% would like access now. Sac-TMT has generated directly relevant evidence in EGFRm NSCLC after EGFR-TKI failure. In OptiTROP-Lung03, sac-TMT improved outcomes versus docetaxel after EGFR-TKI and platinum-based chemotherapy, and in the phase III OptiTROP-Lung04 trial, sac-TMT significantly improved PFS and OS compared with platinum-pemetrexed chemotherapy in patients with EGFR-mutated nonsquamous NSCLC after EGFR-TKI progression [61,62]. Although these data are encouraging, their applicability to Canadian practice will depend on regulatory review, reimbursement, and confirmation of benefit across broader populations.

Priority research directions include: prospective studies defining sequencing after FLAURA2 and MARIPOSA; randomized evaluation of continued EGFR inhibition and local ablative therapy after progression; standardized and clinically validated thresholds for MET amplification; resistance-adapted platform trials for uncommon on-target and bypass mechanisms; and implementation studies evaluating whether rapid molecular technologies can shorten time to treatment without compromising analytic validity. Larger prospective, pan-Canadian studies should also include community and non-academic centres, pathologists, radiation oncologists, patients, and health-system stakeholders to determine whether the proposed pathways are feasible and generalizable across provinces.

## 10. Discussion

Management of EGFRm NSCLC after progression on osimertinib is becoming increasingly complex. The results of expert roundtable discussions and a focused survey highlight that post-osimertinib care is no longer defined by a single standard pathway, but instead by the interaction between evolving evidence, patient-specific factors, and local access to diagnostic testing and therapy. In practice, treatment decisions remain strongly influenced by reimbursement, availability of molecular testing, and the feasibility of obtaining newer agents in a timely manner. At the same time, new first-line regimens such as those studied in FLAURA2 and MARIPOSA are reshaping sequencing and creating new uncertainties regarding the optimal management of progression. Suggested reassessment and treatment pathways are shown in Figure 1 and Figure 2. These pathways are intended as practical decision-support frameworks rather than prescriptive algorithms; they integrate established therapeutic backbones with expert-practice perspectives, access constraints, and areas where evidence remains limited.

### 10.1. Evidence Gaps

Several evidence gaps remain in post-osimertinib EGFRm NSCLC, particularly around treatment sequencing and the role of continuing osimertinib beyond progression. Sequencing after newer first-line regimens such as FLAURA2 and MARIPOSA is not yet well defined, and it remains unclear how to best position amivantamab-based therapy, chemotherapy, rechallenge strategies, or continued EGFR inhibition after these regimens. The evidence for continuing osimertinib with chemotherapy is also still incomplete: clinicians in the questionnaire clearly saw a rationale for preserving CNS control in selected patients, but this remains a pragmatic strategy rather than a universally established standard. Similar uncertainty persists in oligoprogression, where the limited evidence supports local radiotherapy with maintained EGFR inhibition in selected cases, but definitive comparative data remain limited. The ongoing European HALT trial, which evaluates continued TKI therapy with or without SBRT for oligoprogressive disease in mutation-positive NSCLC, may help clarify the role of local ablative therapy in this setting [63]. Finally, in settings such as histologic transformation or uncommon resistance alterations, management remains highly individualized because prospective comparative data are limited and many targeted approaches remain investigational.

### 10.2. Access Gaps

The survey highlighted major access gaps that directly shape real-world care. Although respondents strongly supported rebiopsy and repeat molecular testing at progression, many reported that delays in obtaining biopsies and biomarker results limit the practical utility of this strategy, and routine testing for specific mechanisms such as MET amplification remains inconsistent. Biopsy remains equally important when histologic transformation or other uncommon resistance patterns are suspected. Qualitative responses reinforced the importance of improving access to MET amplification testing and creating a clearer framework for access to targeted therapies in patients with oncogene-driven disease. Transformation to SCLC, although rare, is particularly relevant because it changes management toward histology-directed therapy, most commonly platinum–etoposide, with ongoing uncertainty regarding the role of continuing osimertinib in selected patients.

Access to newer post-osimertinib therapies is an even more prominent issue, particularly for MARIPOSA-2 and MET-directed strategies. Amivantamab-based therapy was noted as highly relevant after osimertinib, yet current reimbursement limitations in some regions restrict real-world use. These findings emphasize that in post-osimertinib EGFRm NSCLC, the identification of a biologically relevant resistance mechanism does not necessarily translate into a clinically actionable treatment pathway. This creates a recurring gap between what is biologically or clinically desirable and what can actually be offered in practice. As a result, even when a potentially actionable resistance mechanism is identified, physicians often still default to platinum-based chemotherapy because it is the most accessible option within the public system. This is most evident when no actionable mutation is present or with transformation to SCLC post-progression. More broadly, even when new regimens are viewed as clinically relevant, their uptake remains strongly shaped by cost-effectiveness, magnitude of benefit, and feasibility within a publicly funded system (Box 1).

Box 1 **Key unmet needs in Canada for EGFRm NSCLC.**

- Short turnaround reflexive testing across all centres.
- Access to MARIPOSA-2 (amivantamab) in second-line therapy across all provinces.
- Access to osimertinib post-progression when adding chemotherapy (to maintain CNS control per COMPEL).
- Approved targeted therapies post-osimertinib (e.g., MET amplification, other EGFR mutations).
- Post-FLAURA2 or MARIPOSA first-line sequencing data.
- Broader access to clinical trials.

Although this survey was Canada-focused, the challenges it identifies are relevant to other countries with a public healthcare system—including those related to limited or variable access to broad molecular testing, variable reimbursement for newer regimens, and difficulty obtaining targeted combinations outside clinical trials. The findings therefore support a broader practical message: post-osimertinib management should remain biologically informed, but it must also be operationally realistic.

Our work has limitations. The work was designed as a practical position paper rather than a formal consensus or systematic-review process, and the evidence and reimbursement landscape continue to evolve rapidly. Additionally, the roundtables, exchanges, and survey involved a relatively small number of experts, and participants were drawn predominantly from Quebec academic centres. The findings may therefore reflect regional referral patterns, testing infrastructure, trial exposure, and reimbursement conditions and should not be interpreted as representative of all Canadian provinces or community settings. However, many of our experts have national leadership roles and our experience is that challenges with drug and biomarker testing access and reimbursement in lung cancer are more similar than different across Canada. Nevertheless, future work should explore these findings in a larger pan-Canadian cohort that includes community and academic clinicians, multiple disciplines, patients, and health-system stakeholders. Despite these limitations, the findings provide a useful contemporary snapshot of how clinicians are navigating post-osimertinib care within the realities of Canadian practice.

## 11. Conclusions

This manuscript provides a practical, contemporary perspective on management after osimertinib in EGFRm NSCLC. The results highlight both emerging areas of agreement and persistent areas of uncertainty. Rebiopsy, chemotherapy, selective continuation of osimertinib, local therapy, and evolving targeted approaches remain key treatment considerations. Real-world access to diagnostic procedures and therapies strongly shapes practice. Although grounded in the Canadian experience, the issues identified are relevant to other countries with publicly funded healthcare systems. Ongoing updates will be needed as the evidence base and reimbursement landscape continue to evolve.

## Figures and Tables

**Table 1:** Key clinical trials examining post-osimertinib treatment that are recruiting in Canada.

NCT	Trial/Intervention	Phase	Population	Primary Endpoint(s)	Est. Completion	Enrollment
NCT06417814	TROPION-Lung15 Dato-DXd + osimertinib vs. Dato-DXd vs. platinum-doublet chemo Anti-TROP2 ADC; biomarker-unselected	3	EGFRm NSCLC; progression on prior osimertinib monotherapy	PFS	Sep 2026 (primary) Sep 2028 (overall)	744
NCT07100080	IZABRIGHT-Lung01 Izalontamab brengitecan (BMS-986507) vs. platinum-pemetrexed HER3-directed ADC	2/3	EGFRm (ex19del/L858R) NSCLC; progression on 3rd-gen EGFR-TKI (incl. osimertinib) as most recent therapy	RP3D (Ph2); PFS by BICR (Ph3)	Sep 2028 (primary) May 2031 (overall)	596
NCT06305754	MK-2870-009 Sacituzumab tirumotecan vs. pemetrexed + carboplatin Anti-TROP2 ADC	3	EGFRm advanced nonsquamous NSCLC; progression on prior EGFR TKI(s)	PFS; OS	Sep 2028 (primary) Jun 2030 (overall)	520
NCT06074588	MK-2870-004 Sacituzumab tirumotecan vs. chemotherapy (docetaxel or pemetrexed) Broader genomic alteration population	3	Previously treated EGFRm or other genomic alteration (ALK, ROS1, BRAF, NTRK, MET ex14, RET) advanced nonsquamous NSCLC	PFS (EGFRm subgroup); OS (EGFRm subgroup)	May 2027 (primary) Mar 2030 (overall)	556
NCT04335292	Osimertinib rechallenge Osimertinib (3rd-line) after 1L osimertinib → 2L chemo sequence Single-arm; Canadian Sponsor	2	EGFRm advanced NSCLC; prior 1L osimertinib + 2L platinum-pemetrexed	ORR (RECIST 1.1)	Jun 2026 (primary) Jun 2027 (overall)	200
NCT06706076	BH-30643 Novel EGFR/HER2 inhibitor targeting resistance mutations Includes acquired EGFR resistance post-osimertinib; early phase	1/2	Advanced NSCLC with EGFR (classical, atypical, ex20ins, acquired resistance) or HER2 mutations; prior standard therapies	DLTs; RP2D (Ph1)/efficacy (Ph2)	Jan 2029 (primary) Jul 2029 (overall)	266

ADC, antibody−drug conjugate; ALK, anaplastic lymphoma kinase; BICR, blinded independent central review; BRAF, B-Raf proto-oncogene; DLT, dose-limiting toxicity; EGFRm, epidermal growth factor receptor-mutated; EGFR-TKI, epidermal growth factor receptor tyrosine kinase inhibitor; ex14, exon 14 skipping; ex19del, exon 19 deletion; ex20ins, exon 20 insertion; HER2, human epidermal growth factor receptor 2; HER3, human epidermal growth factor receptor 3; L858R, leucine-to-arginine substitution at codon 858; MET, mesenchymal–epithelial transition; NCT, national clinical trial; NSCLC, non-small cell lung cancer; ORR, overall response rate; OS, overall survival; PFS, progression-free survival; Ph, phase; RECIST, Response Evaluation Criteria in Solid Tumours; RP2D, recommended phase 2 dose; RP3D, recommended phase 3 dose; RET, rearranged during transfection; ROS1, ROS proto-oncogene 1; NTRK, neurotrophic receptor tyrosine kinase; TROP2, trophoblast cell-surface antigen 2; 1L, first line; 2L, second line; 3rd-gen, third generation.

## Data Availability

The data presented are available on request from the corresponding author due to respondent privacy and ethical restrictions.

## Supplementary Materials

The following supporting information can be downloaded at: https://www.mdpi.com/article/10.3390/curroncol33080465/s1; Table S1: Survey questions and possible answers (multiple choice unless indicated).
