# Supplementary material for: Pragmatic Management of EGFR-Mutant NSCLC After Progression on Osimertinib: Canadian Expert Perspectives
Source: Curr Oncol. 2026 Aug 5;33(8):465. doi: 10.3390/curroncol33080465 (PMC13510353; doi:10.3390/curroncol33080465)
Supplement: Supplementary file 1 [file curroncol-33-00465-s001.zip › curroncol-4419797-supplementary.pdf]

Supplemental Table S1. Survey questions and possible answers (multiple choice unless indicated)

Section	Question	Possible answers
First-line treatment perspective	**Q1.** First-line treatment for EGFR-mutated lung cancer has expanded over the past year. In your opinion, what proportion of patients will receive each of the following treatments?	For each regimen (Osimertinib, FLAURA2, MARIPOSA): 0–9%, 10–24%, 25–49%, 50–74%, 75–100%
First-line treatment perspective	**Q2.** Aside from age and comorbidities, for which patients would you still consider osimertinib?	No brain metastases; Physician preference for ease of administration / toxicity management; Low tumour burden; Patient prioritizes quality of life; Patient preference for ease of administration / toxicity management
First-line treatment perspective	**Q3.** After FLAURA-2, what would be your preferred treatment regimen at progression, assuming access were not an issue?	Docetaxel; Platinum-doublet rechallenge and continue osimertinib; Clinical trial, if possible; MARIPOSA-2
Rebiopsy	**Q1.** Do you still favor rebiopsy for all patients at progression on osimertinib?	Liquid biopsy only; No, I do not perform rebiopsy; No, I biopsy only in selected situations (case by case); Tissue biopsy, if possible; Tissue + liquid biopsy
Rebiopsy	**Q2.** What is the turnaround time for obtaining biomarker/NGS results in your center?	5 weeks or more; 3 weeks; 4 weeks; 2 weeks or less
Rebiopsy	**Q3.** Do you also request testing for MET amplification (for example by FISH) in addition to NGS?	Yes; No

Section	Question	Possible answers
Rebiopsy	**Q4.** Can delays in obtaining the biopsy and the results be a barrier to requesting a biopsy?	Yes; No
Rebiopsy	**Q5** *(second Q4 in the slides)*. With MARIPOSA-2, do you find that fewer rebiopsies are being requested, given the MET inhibition provided by amivantamab?	Yes, fewer biopsies are requested; No, I still request rebiopsy at progression
Treatment at progression without an actionable target	**Q1.** If access were not a problem, what would be your preferred treatment after osimertinib?	Chemotherapy alone (platinum doublet); Clinical trial; Continue osimertinib + add chemotherapy (platinum doublet); MARIPOSA-2
Treatment at progression without an actionable target	**Q2.** Do you think it is important to advocate for continuation of osimertinib beyond progression together with chemotherapy?	No, we have better treatment options; Maybe, as this could be useful for some patients; Yes, it is important to maintain CNS inhibition and have access to osimertinib in this context
Treatment at progression without an actionable target	**Q3.** What are your impressions of the MARIPOSA-2 regimen?	I have used it and did not see added value; I have not used it; I have used it; benefits are limited; I have used it; it is useful and relevant
Treatment at progression without an actionable target	**Q4.** What are your impressions of the INESSS decision (negative recommendation) regarding MARIPOSA-2?	Free-text comments
Treatment at progression without an actionable target	**Q5.** Do you sometimes use immunotherapy as a last resort after chemotherapy?	Yes, I have proposed immunotherapy if PD-L1 is high (50%+); Yes, I propose immunotherapy regardless of PD-L1 expression; I do not use immunotherapy in EGFR-mutant disease, even as a last resort

Section	Question	Possible answers
Oligoprogressi on	**Q1.** Does radiotherapy for oligoprogression remain important in the era of new potential therapies?	Yes, I want to keep osimertinib as long as possible and consider radiotherapy as needed**;** No, I am more inclined to change treatment if I can obtain amivantamab or another systemic therapy
Anti-MET therapy	**Q1.** Regarding targeted anti-MET therapies after osimertinib, which treatment do you consider the most promising?	Tepotinib + osimertinib**;** Teliso-V + osimertinib**;** Savolitinib + osimertinib**;** MARIPOSA-2, i.e., amivantamab + chemotherapy
Anti-MET therapy	**Q2.** Have you been able to obtain a targeted anti-MET therapy combined with osimertinib outside of a research protocol?	Yes, via PAS (special access / Santé Québec)**;** Yes, via company compassionate program**;** Yes, the patient paid out of pocket**;** No
Small-cell lung cancer transformation after osimertinib	**Q1.** Which treatment do you favor when transformation occurs?	Platinum–etoposide + immunotherapy**;** I do not biopsy, so I do not know whether transformation is present**;** Platinum–etoposide + osimertinib**;** Platinum–etoposide
Future of post-progression treatment	**Q1.** Are you satisfied with the number and variety of clinical trials open in Quebec for EGFR-mutated lung cancer?	Yes, there are enough**;** No, there should be more
Future of post-progression treatment	**Q2.** What do you think about anti-TROP2 agents in EGFR-mutated lung cancer, particularly datopotamab deruxtecan (TROPION-Lung05)?	Anti-TROP2 agents are not relevant in this population**;** Dato-DXd is promising after osimertinib + chemotherapy; I would like access now**;** Dato-DXd is promising after osimertinib + chemotherapy; I will wait for ongoing studies before taking a position

Section	Question	Possible answers
Future of post-progression treatment	**Q3.** What do you think about anti-TROP2 agents in EGFR-mutated lung cancer, particularly SAC-TMT (OptiTROP-Lung04)?	Anti-TROP2 agents are not relevant in this population**;** SAC-TMT is promising after osimertinib; I would like access now**;** SAC-TMT is promising after osimertinib; I will wait for ongoing studies before taking a position
Future of post-progression treatment	**Q4.** What do you think about the data for ivonescimab +	
